# Involuntary Commitment and Treatment in the Nordic Countries: A Comparative Analysis

**DOI:** 10.1192/j.eurpsy.2026.11642

**Published:** 2026-07-31

**Authors:** G. Mijaljica

**Affiliations:** 1 St Charles Hospital, Central and North West London NHS Foundation Trust, London, United Kingdom; 2 Transcultural Centre, Region Stockholm, Stockholm, Sweden

## Abstract

**Introduction:**

Involuntary commitment to a psychiatric institution is regulated by national legislation and entails particular scrutiny, as it involves, among other concerns, the deprivation of liberty. Although there is broad consensus that involuntary commitment and treatment should be kept to a minimum, research indicates relatively high rates of such practices in some Nordic countries. Furthermore, legislation governing involuntary commitment and treatment shows considerable variation, even within the Nordic region.

**Objectives:**

This comparative analysis examines the key aspects of legislation on involuntary commitment and treatment in Denmark and Greenland, Finland, Norway, Sweden, and Iceland.

**Methods:**

Laws regulating involuntary commitment and treatment in the Nordic countries were identified and analysed with a focus on key definitions and provisions concerning consent and capacity, deprivation of liberty, involuntary treatment and interventions, judicial and other review mechanisms, and community treatment orders.

**Results:**

While there are similarities in core legal definitions and procedures, substantial differences remain - for example, in the formal decision-making process regarding deprivation of liberty, the initiation of involuntary treatment, and the mechanisms of legal and judicial review.

**Conclusions:**

These differences, particularly in definitions, decision-making processes, and review mechanisms, suggest that common values are implemented in diverse ways, raising important questions about ethics, rights protection, and best practices. Further research is needed to better understand the ethical and legal aspects of these processes, as well as the cultural dimensions that may contribute to different approaches to involuntary commitment.

**Disclosure of Interest:**

None Declared

